# Intranasal dexmedetomidine with morphine or tramadol: A comparative study of effects on alfaxalone requirements for anesthesia in cats

**DOI:** 10.14202/vetworld.2023.1201-1208

**Published:** 2023-06-04

**Authors:** Kewvaree Hommuang, Nattika Koatsang, Suttiporn Srikullabutr, Panpicha Sattasathuchana, Naris Thengchaisri

**Affiliations:** 1 Department of Clinical Sciences and Public Health, Faculty of Veterinary Sciences, Mahidol University, Salaya, Phutthamonthon District, Nakhon Pathom, 73170, Thailand; 2 Surgery Unit, Kasetsart University Veterinary Teaching Hospital, Bangkok, 10900, Thailand; 3 Department of Companion Animal Clinical Science, Faculty of Veterinary Medicine, Kasetsart University, Bangkok, 10900, Thailand; 4 Department of Companion Animal Clinical Sciences, Faculty of Veterinary Medicine, Kasetsart University, Bangkok, 10900, Thailand

**Keywords:** alfaxalone, dexmedetomidine, intranasal, morphine, tramadol

## Abstract

**Background and Aim:**

Intranasal (IN) sedatives provide a non-invasive route for premedication drug administration. This study compared the cardiorespiratory and sparing effects of IN dexmedetomidine combined with morphine (DM) or tramadol (DT) on alfaxalone requirements for anesthesia induction in cats.

**Materials and Methods:**

Twenty-four cats were randomly assigned to three groups: Dexmedetomidine combined morphine (IN dexmedetomidine 20 μg/kg plus 0.2 mg/kg morphine), DT (IN dexmedetomidine 20 μg/kg plus 1 mg/kg tramadol), or control (no premedication). The intravenous dose of 1% alfaxalone for endotracheal intubation was recorded with sedation scores, cardiorespiratory parameters (heart rate and respiration rate), and side effects.

**Results:**

Both DM and DT were associated with significantly higher sedation scores than baseline, and sedation scores were found to be highest 20 min after premedication. Sedation scores were comparable between DM and DT groups. Side effects, including hypersalivation, vomiting, and pupillary dilation, were observed in the DM and DT groups. The dosage of alfaxalone required in the DM group (1.5 ± 0.3 mg/kg) was comparable to that of the DT group (2.0 ± 0.6 mg/kg, p = 0.0861), and both groups required significantly less alfaxalone than the control group (3.0 ± 0.6 mg/kg; p < 0.01). Heart and respiratory rates were comparable between the DM and DT groups. Duration of anesthesia in the control group (11 ± 4 min) was significantly shorter than in the DM (29 ± 5 min, p = 0.0016) and DT (38 ± 14 min, p < 0.001) groups.

**Conclusion:**

Intranasal administration of DM or DT produces good sedation and offers an alternative, non-invasive route for cats undergoing general anesthesia.

## Introduction

Intranasal administration is easy, non-invasive, and safe to deliver analgesia and reduce stress. Intranasal (IN) administration of anesthetic drugs has been used in multiple species, including humans [1, 2], dogs [3, 4], and cats [5, 6]. Recently, we demonstrated that dexmedetomidine, a potent alpha-2 adrenoreceptor agonist [7], through IN administration provided an alternative route for sedation and reduced the dosage of propofol required for anesthesia induction in cats [6]. Drug delivery through the IN route effectively targets the brain to provide sedation and premedication [8].

Neuroleptanalgesia involves the combination of an opioid analgesic and a sedative or tranquilizer. The benefits are reduced adverse cardiopulmonary effects compared with the drugs administered individually [9]. Dexmedetomidine is a selective α2-adrenergic receptor agonist that acts centrally to produce sedative, anxiolytic, and analgesic effects [2, 7]. Intranasal dexmedetomidine is absorbed through the nasal mucosa, rapidly reaching the systemic circulation, and crossing the blood–brain barrier [4, 6]. Once in the brain, dexmedetomidine binds to α2-adrenergic receptors located in the locus coeruleus, a region that regulates sleep and arousal. The activation of these receptors reduces norepinephrine release, leading to sedation, anxiolysis, and analgesia [1, 2, 7]. Dexmedetomidine can be administered alone or in combination with opioids. Morphine is a mu-opioid receptor agonist that produces potent analgesia, as shown by its IN administration in humans [10]. Tramadol is a synthetic opioid agonist of the mu-opioid receptor that can produce analgesia and sedation in cats [11]. Intranasal tramadol administration in rodents has been found to produce a faster onset of action and better analgesia over time than oral treatment [12]. Alfaxalone is a synthetic neuroactive steroid that modulates the gamma-aminobutyric acid A receptor causing depression in the nervous system [13]. Alfaxalone is considered a clinically effective anesthesia induction agent and is commonly used to induce general anesthesia in cats [14]. However, the effectiveness of neuroleptanalgesia through IN administration of dexmedetomidine with morphine (DM) or tramadol (DT) has not been demonstrated in cats.

The present study hypothesizes that IN administration of dexmedetomidine combined with either morphine or tramadol could provide effective sedation and reduce the dose of alfaxalone required for anesthesia induction in cats. Sedation scores, cardiorespiratory effects, side effects after IN administration, and dose-sparing effects on alfaxalone requirements for anesthesia induction in cats were also examined.

## Materials and Methods

### Ethical approval and informed consent

Approval was obtained from the Kasetsart University Animal Committee (ID number: ACKU65-VET-054) and the Ethical Review Board of the Office of the National Research Council of Thailand (NRCT license U1-00500-2558). Written consent was obtained from all cat owners, and all procedures in this clinical trial complied with the Kasetsart University Institutional Animal Care and Use Standards.

### Study period and location

The study was conducted from June to August 2022. Twenty-four cats visiting the dental unit at the Kasetsart University Veterinary Teaching Hospital in Bangkhen, Thailand, were enrolled in the present study.

### Animals

Twenty-four client-owned cats (12 males and 12 females) aged 2–7 years (3.3 ± 0.9) and weighing 2.5–7.0 kg (4.4 ± 1.0) were randomly selected and enrolled in this study for dental scoring and examination. All cats underwent a physical examination and received complete blood counts and serum biochemistry (alanine transaminase, alkaline phosphatase, blood urea nitrogen, creatinine, and albumin). Cats with abnormal blood test results and anatomical abnormalities, particularly those with extremely flat faces or brachycephalic skulls, were excluded from the study. Cats were classified into the American Society of Anesthesiologists (ASA) physical status of ASA I or II. The cats were equally and randomly assigned to receive one of three treatments. All cats underwent a fasting period of approximately 8 h in accordance with the new American Animal Hospital Association and World Small Animal Veterinary Association guidelines. Water and food were withheld from the cats after midnight before the start of the clinical trial the following morning.

### Study protocol

Each cat was placed in a quiet, dim room to acclimate to the environment and staff for 10 min. The observer then performed a physical examination before starting the experiment (T_0_). A veterinarian performed a general physical examination, including evaluation of thoracic auscultation, respiratory rate, heart rate, pulse palpation, mucous membrane color, capillary refill time, and body condition score. The temperament of the cats was evaluated on a descriptive scale (before and after sedation) [15]. A descriptive temperament scale ranging from 0 to 2 was used: 0, cat does not mind physical examination; 1, cat is scared or nervous during physical examination; and 2, cat is aggressive.

Cats were randomly allocated into three groups of four males and four females each. Group 1 (DM), dexmedetomidine (20 µg/kg, Dexdomitor^®^; Virbac [Thailand] Co., Ltd., Thailand) plus morphine (0.2 mg/kg, M&H Manufacturing Co., Ltd., Thailand), administered through the IN route, followed by alfaxalone titrated to effect (Jurox, UK). Group 2 (DT), dexmedetomidine (20 µg/kg) plus tramadol (1 mg/kg, Harson Laboratories, Akota, Baroda, India), administered through IN followed by alfaxalone. Group 3, Control, alfaxalone 1 mg/kg delivered intravenously (IV) without premedication. Before the drug administration, each cat was restrained in sternal recumbency. The drugs were administered equally in the DM and DT groups by dividing the final volume into both nostrils using a 1-mL syringe. The cat’s head was raised approximately 30 degrees while receiving the IN drugs. Total drug volume was also recorded for DM and DT groups. The respiratory rate, heart rate, and rectal temperature were recorded every 10 min after T_0_. The same veterinarian recorded the sedation scores to help reduce interpersonal variation.

Sedation scores were assessed and recorded at time points 0 (T_0_), 5 (T_5_), 10 (T_10_), 15 (T_15_), and 20 (T_20_) min after dexmedetomidine administration using a numeric descriptive sedation scale [1] and composite numeric rating scale [15]. The numeric descriptive sedation scale [1] was scored as follows: 0, normal; 1, mild ataxia; 2, moderate sedation; and 3, deep sedation. The composite numeric rating scale [15] ranged from 0 to 10, where 0 is normal and 10 is deep sedation. The composite numeric rating is the sum of the individual scores of four different assessments: posture (0–4), response to clipper sounds (0–2), response to clipping (0–2), and response to restraint (0–2). A single-experienced veterinarian performed the evaluations. During the sedation observation period, side effects (vomiting, hyperthermia, or pupillary dilation) were also recorded. Pupillary diameter in this study was measured using a Vernier caliper (Louisware; S.P.S. Lab Co., Ltd., Thailand).

Twenty minutes after IN administration (for cats in the DM and DT groups) or without IN administration (for cats in the control group), the cat was gently restrained to place a 24-gauge IV catheter in the cephalic vein for induction. Anesthesia was induced with IV alfaxalone beginning with a loading dose of 1 mg/kg followed by titration of 0.5 mg/kg doses every 45 s until there was loss of jaw tone and no or minimal gagging. The total dose of IV alfaxalone administered to achieve endotracheal intubation was recorded. Before endotracheal tube (ETT) insertion, xylocaine 10% spray (Lidocaine 10 mg/puff; AstraZeneca AB, Södertälje, Sweden) was applied to desensitize the larynx. A Miller laryngoscope (Roester, Germany) was used for ETT insertion, and its cuff was inflated to 20 cm H_2_O using a pressure gauge (Hi-LoÔ Hand Pressure Gauge; Covidien, USA). The ETT was secured with gauze. After orotracheal intubation, the cats were attached to a non-rebreathing system to supply 100% oxygen (250 mL/kg/min) during dental scaling. Lactated Ringer’s solution (General Hospital Products Public Co., Ltd., Thailand) was administered IV at 5 mL/kg/h until extubation.

After intubation, heart rate, respiratory rate, hemoglobin oxygen saturation, rectal temperature, palpebral reflex response, and pedal reflex response were continuously measured every 10 min using a multiparameter physiological monitor (Datex-Ohmeda CARESCAPE Multifunctional Anesthesia Monitor; GE Healthcare Finland). Variables were recorded at T_0_, T_10_, T_20_, T_30_, T_40_, T_50_, and T_60 min_. Sixty minutes after intubation, atipamezole hydrochloride (Antisedan^®^; Virbac Co., Ltd.) was administered at a half-dose (100 μg/kg) relative to dexmedetomidine to facilitate recovery. Extubation was performed when the cat recovered its gag reflex. Side effects, including hyperthermia and hypersalivation, were recorded. Anesthesia duration (time elapsed from alfaxalone administration to extubation) and procedure success (defined as the cats being sufficiently anesthetized until the return of airway reflexes) were also recorded.

### Recovery

All cats were monitored for 1 h after extubation for upper respiratory airway discomfort, including stridor, coughing, retching, and hoarse voice. Recovery time (time from extubation to the cat being capable of sternal recumbency) was recorded for each cat. After full recovery from general anesthesia, the cats were returned to their owners. Owners were instructed to observe and record any abnormal signs such as coughing, vomiting, and abnormal posture during the first 24 h at home.

### Statistical analysis

STATA12 (StataCorp, College Station, TX, USA) and GraphPad Prism version 6 (GraphPad Software, Inc., La Jolla, CA, USA) were used to estimate the required sample size using Student’s t-test with a power of 80% and an alpha error of 0.05 to detect differences in the required dose of alfaxalone (approximately 0.5 mg/kg). All data were tested for normality using the Shapiro–Wilk test. Average and composite sedation scores between the DM and DT groups at different time points (T_0_, T_5_, T_10_, T_15_, and T_20_ after drug administration) were tested using a two-way analysis of variance followed by Tukey’s multiple comparison test. Associations between drug administration and categorical data, including side effects (vomiting and pupillary dilation), body condition score, ASA status, and temperature, were determined using Fisher’s exact test. Other variables, including dosage of alfaxalone, respiratory rate, heart rate, hemoglobin oxygen saturation, and body temperature, were summarized across the three groups as the mean ± standard deviation. A two-way analysis of variance was used to compare the physiological variables between the three groups. The significance level was set at p < 0.05.

## Results

There were no statistically significant differences among the three groups for age, sex, body weight, body condition score, ASA status, or breed. The cats’ temperaments during pre-anesthetic examination were also comparable among the three groups (Table-1).

**Table-1:** Characteristics of cats in the DM and DT groups.

Characteristic	DM	DT	Control
Mean age ± SD (months)	45.0 ± 8.5	37.5 ± 9.5	38.3 ± 12.0
Sex (n)			
Female	4	4	4
Male	4	4	4
Mean body weight ± SD (kg)	4.1 ± 1.0	4.0 ± 1.0	4.3 ± 1.1
Median body condition score	3.2 ± 0.8	2.8 ± 0.6	3.0 ± 0.2
Median ASA status	1.4 ± 0.5	1.6 ± 0.5	1.5 ± 0.5
Breed (n)			
DSH	8	8	7
Other	0	0	1
Median temperament (range)	0.5 ± 0.5	0.9 ± 0.8	0.4 ± 0.7

Control=No intranasal administration, ASA=American Society of Anesthesiologists, DM=Dexmedetomidine combined morphine, DSH=Domestic shorthair, DT=Dexmedetomidine combined tramadol, SD=Standard deviation

**Figure-1:**
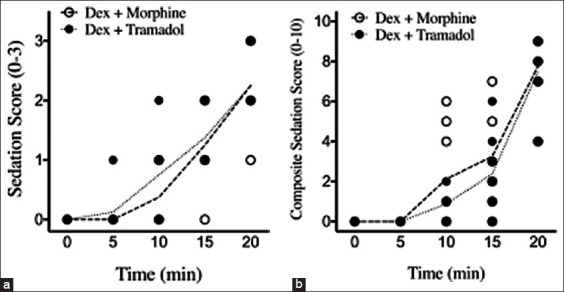
Median sedation scores and median composite sedation scores in cats after premedication. (a) There was no significant difference in sedation scores (0–3) between the DM and DT groups (p > 0.05). (b) There was no significant difference in composite sedation scores (0–10) between the DM and DT groups (p > 0.05). Findings are based on a two-way repeated measures analysis of variance with Tukey’s multiple comparison tests. DM=Dexmedetomidine combined morphine, DT=Dexmedetomidine combined tramadol.

All cats exhibited significant sedation 20 min after the DM or DT protocol was administered. Median sedation scores of cats receiving DM were comparable to those in the DT group at multiple time points (T_5_, T_10_, T_15_, and T_20_). There was no statistically significant difference between the two groups (Figure-1a). Median composite sedation scores of cats receiving DM were comparable to those of cats in the DT group at multiple time points (T_5_, T_10_, T_15_, and T_20_). There was no statistically significant difference between the two groups (Figure-1b).

**Figure-2:**
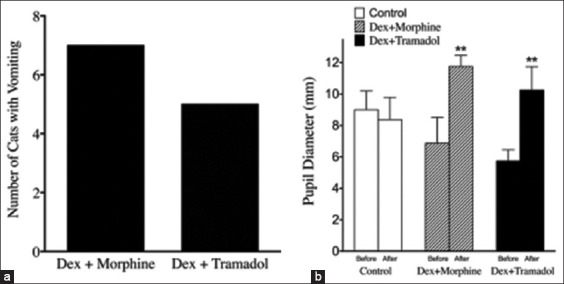
Side effects identified in cats after premedication. (a) Number of cats with vomiting after premedication in the DM and DT groups. (b) Pupil diameter measurement in cats after premedication in the DM and DT groups compared with the control group. **p < 0.01 versus control group. DM=Dexmedetomidine combined morphine, DT=Dexmedetomidine combined tramadol.

Side effects following IN administration in the DM and DT protocols are shown in Figure-2. Nausea and drooling were present in 87.5% of cats in the DM group and 100% of cats in the DT group with no statistically significant difference between the two groups. Vomiting was observed in 87.5% of cats in the DM group and in five cats (62.5%) in the DT group (Figure-2a). Pupillary dilation was observed in the DM and DT groups following IN administration (Figure-2b). The pupillary diameters of cats in the control group were unchanged (p > 0.05; Figure-2b).

**Figure-3:**
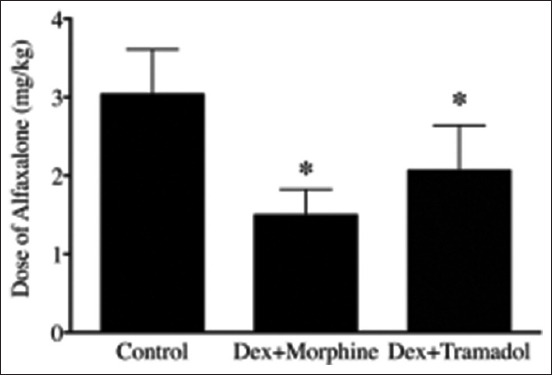
Dose of alfaxalone required for endotracheal intubation in cats. The DM and DT groups required a lower alfaxalone dosage compared with the control group (no premedication). *p < 0.05 versus control, Student’s t-test. DM=Dexmedetomidine combined morphine, DT=Dexmedetomidine combined tramadol.

**Figure-4:**
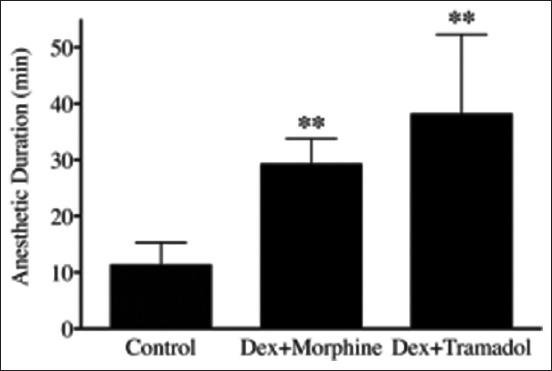
Anesthetic duration after alfaxalone induction. Both the DM and DT groups had longer anesthetic duration compared with the control group. **p < 0.01 versus control, Student’s t-test. DM=Dexmedetomidine combined morphine, DT=Dexmedetomidine combined tramadol.

Anesthetic-sparing effects of the IN DM and DT protocols were evaluated using alfaxalone for anesthetic induction. The amount of alfaxalone required for endotracheal intubation in the control group (3.0 ± 0.6 mg/kg; Figure-3) was higher than in the DM group (1.5 ± 0.3 mg/kg, p < 0.001) and the DT group (2.0 ± 0.6 mg/kg, p = 0.0024). However, there was no significant difference in alfaxalone dosage between the DM and DT groups (p = 0.0861; Figure-3). Anesthesia duration following alfaxalone was also compared among the three groups. Anesthesia duration for the control group (11 ± 4 min) was significantly shorter than that for the DM group (29 ± 5 min, p = 0.0016) and the DT group (38 ± 14 min, p < 0.001). However, there was no significant difference in duration between the DM and DT groups (p = 0.1376; Figure-4).

**Figure-5:**
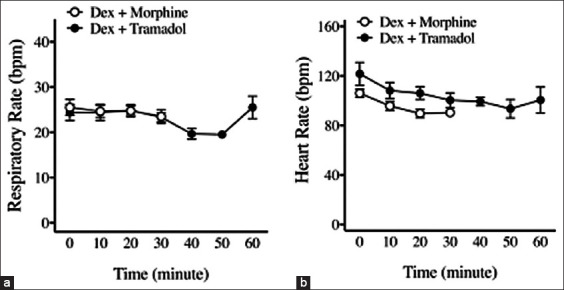
Respiratory rate and heart rate in cats receiving premedication and intravenous alfaxalone anesthesia. (a) Comparable respiratory rate was found in both DM and DT groups. (b) Comparable heart rate was found in both DM and DT groups. Two-way repeated measures analysis of variance with Tukey’s multiple comparison test. DM=Dexmedetomidine combined morphine, DT=Dexmedetomidine combined tramadol.

During maintenance anesthesia, respiratory (Figure-5a) and heart rates (Figure-5b) were comparable between the DM and DT groups. All parameters were found within normal limits.

## Discussion

The present study evaluated the efficacy of IN administration of DM and DT as premedications and compared the dose-sparing effects of alfaxalone on the requirements for anesthesia induction in cats. There was no clinically significant difference between IN DM or DT for sedation. Intranasal premedication with either DM or DT protocols decreased the amount of alfaxalone required for endotracheal intubation compared with the control group. There was no significant difference in side effects, alfaxalone dosage, or anesthesia duration between the DM and DT groups.

This study also found that the sedative effects of the IN DM and DT protocols did not differ. All cats had moderate sedation 20 min following IN administration of either DM or DT. This was similar to previous studies finding that IN dexmedetomidine in cats resulted in deep sedation 20 min after drug administration [6]. In contrast, sedation onset when using a dexmedetomidine-ketamine-morphine combination administered through the IN route in young dogs was 2–10 min [3]. This suggests that drug delivery through the nasal mucosa in dogs may be more effective than in cats. However, several studies have hypothesized that this direct pathway exists for some drugs [16–18]. In addition, the direct nose-to-brain pathway depends on multiple factors such as species, individual variation, drug dosage, health status, and device delivery factors that may influence drug absorption through the nasal mucosa [2, 16, 18].

Vomiting is a common side effect of dexmedetomidine and opioid administration in cats. Dexmedetomidine, an alpha-2-adrenergic agonist, and opioids cause vomiting by activating receptors in the chemoreceptor trigger zone in cats [19, 20]. Martin-Flores *et al*. [19] reported that the incidence of emesis from a combination of dexmedetomidine and morphine administered preoperatively to healthy cats was 59%. In the present study, vomiting following IN administration was observed in seven cats in the DM group (87.5%) and five cats in the DT group (62.5%). These results may support the notion that nasal drug delivery is an effective route for targeting the central nervous system [8, 16, 18]. Furthermore, the most common opioid side effects are nausea, vomiting, constipation, and drowsiness [10]. In the present study, nausea and drooling were observed in seven cats in the DM group (87.5%) and eight cats in the DT group (100%) during the sedation phase.

Pupillary dilation was another side effect observed in this study. All cats in the DM and DT groups had pupillary dilation 20 min after IN administration. We hypothesize that the opioid administration caused this mydriasis. Many studies have investigated opioid-induced pupillary changes in animals [21–23]. In cats, morphine disrupts parasympathetic innervation of the iris through its interaction with opiate receptors, resulting in mydriasis and a decrease in the pupillary light reflex [21–23]. This contrasts with opioid-induced miosis in humans [24]. Light-induced pupillary changes occur because the pathway involves optic and oculomotor nerves constricting the pupils following light exposure. Light-sensitive neurons have been recorded from the pretectal and oculomotor nucleus regions of this pathway in cats [24]. Moreover, morphine increases the spontaneous firing rate of all light-sensitive neurons, including the nucleus of the third nerve. Another pathway is the release of catecholamines, primarily from the adrenal gland, which acts directly as a peripheral sympathetic input to the iris. Although morphine-activated parasympathetic input to the iris should produce pupillary constriction, this effect is antagonized by the increased level of catecholamines resulting in mydriasis in cats [22]. Tramadol administration can also cause mydriasis in cats [23, 25], and as in humans, it may affect the stimulation of opioid receptors [26] due to the inhibition of serotonin and norepinephrine reuptake [27]. Thus, DM and DT premedication should not be used in glaucoma or cataract surgery because they induce mydriasis. Respiratory depression, which can occur due to the use of an opioid substance, was not observed after IN morphine or tramadol administration in this study.

If alpha-2-agonists and opioids are used together, certain effects, including analgesia and sedation, may be improved [28]. The benefits of this combination include reduced adverse cardiopulmonary effects compared with the drugs being administered individually. Using these drugs together also significantly helps reduce the dose of induction agents [28, 29]. In this study, cats in the DM group (1.5 ± 0.3 mg/kg) and the DT group (2.0 ± 0.6 mg/kg) required significantly less alfaxalone for tracheal intubation after IN premedication compared with those in the control group (3.0 ± 0.6 mg/kg). In dogs, the sparing effects of morphine and tramadol have been demonstrated previously [30, 31]. These medications can reduce anesthetic requirements in dogs [30]. However, in this study, there was no significant difference in alfaxalone dosage between the DM and DT groups. The previous studies have reported that the IV dose of alfaxalone required to achieve endotracheal intubation in cats was decreased using premedication [32, 33]. This study found that IN administration of DM could reduce the alfaxalone dosage required for intubation by almost 50% (1.5 ± 0.3 mg/kg) with a 33% reduction (2.0 ± 0.6 mg/kg) for DT premedication. This marked reduction may indicate the benefits of IN premedication, which produces a sedative effect and a lower alfaxalone requirement for anesthesia induction compared with no premedication. During the induction phase, adverse effects of alfaxalone administration, such as apnea and pain during injection, were not observed. This is similar to the findings of other studies [34, 35].

During anesthesia maintenance, the heart rate and respiratory rate after receiving IV alfaxalone did not differ between the DM and DT groups. This study found that anesthesia duration until extubation in the DM (29 ± 4 min) and DT (38 ± 14 min) groups were longer than in the control group (11 ± 4 min). This prolonged anesthesia duration may have been due to the residual sedative and analgesic effects of morphine or tramadol. These drugs were able to provide effective pain relief and minimize any stimulation from pain, thus resulting in better tolerance of the ETT as the cats were regaining consciousness [32]. Furthermore, atipamezole was applied after endotracheal extubation in the DM and DT groups. Nonetheless, there was no significant difference in anesthetic duration between the DM and DT groups.

Intranasal administration is a non-invasive method to rapidly deliver drugs directly from the nasal mucosa to bypass the blood–brain barrier and target therapeutics directly to the central nervous system using pathways along the olfactory and trigeminal nerves [8, 16]. This study found that DM or DT can reach the brain after IN administration, possibly because the drugs are small, lipophilic molecules that distribute to well-perfused tissues such as the brain [6, 8, 12, 16]. This trial found that IN administration of DM and DT was easy to perform, although it caused nausea and drooling in some cats. Intranasal administration is an alternative route for drug administration that does not involve using a needle; therefore, it can be used for at-home drug administration, especially for pain control and stress reduction.

Because there is little information regarding the application of premedication using the IN route, several limitations were encountered in the present study, including a limited volume of drugs for administration and a lack of a titration strategy for optimal dosing. The effects of premedication on patient blood pressure were also not evaluated. Therefore, cardiovascular side effects of IN dexmedetomidine with morphine or tramadol could not be fully evaluated. Moreover, we did not induce any significant pain during the procedure; therefore, the analgesic effects of IN dexmedetomidine with morphine or tramadol require further evaluation.

## Conclusion

Intranasal administration of DM or DT produces good sedation. Some side effects may occur, such as hypersalivation, vomiting, and pupillary dilation. The sparing effects of alfaxalone for anesthesia induction were comparable between the DM and DT approaches. Thus, IN administration of DM and DT provides an alternative, non-invasive route for cats undergoing general anesthesia. Because this study did not induce significant pain during the procedure, the analgesic effects of IN dexmedetomidine along with morphine or tramadol require future evaluation.

## Authors’ Contributions

KH, NK, and NT: Conceptualization and methodology. KH, NK, SS, and NT: Conducted experiments. PS and NT: Data analysis. KH and NK: Writing-original draft preparation. KH, NK, SS, PS, and NT: Writing-review and editing. KH and NT Funding acquisition. All authors have read, reviewed, and approved the final manuscript.

## References

[1] Yuen V.M, Irwin M.G, Hui T.W, Yuan M, Lee L.H.Y (2007). A double-blind, crossover assessment of the sedative and analgesic effects of intranasal dexmedetomidine. Anesth. Analg.

[2] Iirola T, Vilo S, Manner T, Aantaa R, Lahtinen M, Scheinin M, Olkkola K.T (2011). Bioavailability of dexmedetomidine after intranasal administration. Eur. J. Clin. Pharmacol.

[3] Canpolat I, Karabulut E, Cakir S (2016). The efficacy of intranasal administration of dexmedetomidine, ketamine and morphine combination to young dogs. Int. J. Curr. Res.

[4] Santangelo R, Harel M, Fourel I, Micieli F, Cataldi M, Segard-Weisse E, Portier K (2019). Intranasal dexmedetomidine in healthy beagles:An echocardiographic and pharmacokinetic/pharmacodynamic study. Vet. J.

[5] Margani M, Akbarinejad V, Bagheri M (2015). Comparison of intranasal and intramuscular ketamine-midazolam combination in cats. Vet. Anaesth. Analg.

[6] Hommuang K, Sattasathuchana P, Thengchaisri N (2022). Effects of intranasal and intramuscular dexmedetomidine in cats receiving total intravenous propofol anesthesia. Vet. World.

[7] Ansah O.B, Raekallio M, Vainio O (1998). Comparison of three doses of dexmedetomidine with medetomidine in cats following intramuscular administration. J. Vet. Pharmacol. Ther.

[8] Pansini V, Curatola A, Gatto A, Lazzareschi I, Ruggiero A, Chiaretti A (2021). Intranasal drugs for analgesia and sedation in children admitted to pediatric emergency department:A narrative review. Ann. Tranal. Med.

[9] Bissonnette B, Swan H, Ravussin P, Un V (1999). Neuroleptanesthesia:Current status. Can. J. Anaesth.

[10] Kendall J.M, Reeves B.C, Latter V.S (2001). Multicentre randomised controlled trial of nasal diamorphine for analgesia in children and teenagers with clinical fractures. BMJ.

[11] Zhang H, Zhao Y, Wang X, Zhang Q (2014). Bioavailability of tramadol hydrochloride after administration via different routes in rats. Biopharm. Drug Dispos.

[12] Albertson T.E, Walby W.F, Joy R.M (1992). Modification of GABA-mediated inhibition by various injectable anesthetics. Anesthesiology.

[13] Tamura J, Oyama N, Fukui S, Yamashita K (2021). Comparison of the anesthetic effects between 5 mg/kg of alfaxalone and 10 mg/kg of propofol administered intravenously in cats. J. Vet. Med. Sci.

[14] Bhalla R.J, Trimble T.A, Leece E.A, Vettorato E (2017). Comparison of intramuscular butorphanol and buprenorphine combined with dexmedetomidine for sedation in cats. J. Feline Med. Surg.

[15] Evangelista M.C, Silva R.A, Cardozo L.B, Kahavegian M.A.P, Rossetto T.C, Matera J.M, Fantoni D.T (2014). Comparison of preoperative tramadol and pethidine on postoperative pain in cats undergoing ovariohysterectomy. BMC Vet. Res.

[16] Dhuria S.V, Hanson L.R, Frey W.H (2010). Intranasal delivery to the central nervous system:Mechanisms and experimental considerations. J. Pharm. Sci.

[17] Di Salvo A, Conti M.B, Nannarone S, Bufalari A, Giorgi M, Moretti G, Marenzoni M.L, Rocca G.D (2020). Pharmacokinetics and analgesic efficacy of intranasal administration of tramadol in dogs after ovariohysterectomy. Vet. Anaesth. Analg.

[18] Crowe T.P, Hsu W.H (2022). Evaluation of recent intranasal drug delivery systems to the central nervous system. Pharmaceutics.

[19] Martin-Flores M, Sakai D.M, Learn M.M, Mastrocco A, Campoy L, Boesch J.M, Gleed R.D (2016). Effects of maropitant in cats receiving dexmedetomidine and morphine. J. Am. Vet. Med. Assoc.

[20] Nagore L, Soler C, Gil L, Serra I, Soler G, Redondo J.I (2012). Sedative effects of dexmedetomidine, dexmedetomidine-pethidine and dexmedetomidine-butorphanol in cats. J. Vet. Pharmacol. Ther.

[21] Pickworth W.B, Sharpe L.G (1985). Morphine-induced mydriasis and inhibition of pupillary light reflex and fluctuations in the cat. J. Pharmacol. Exp. Ther.

[22] Wallenstein M.C, Wang S.C (1979). Mechanism of morphine-induced mydriasis in the cat. Am. J. Physiol.

[23] Schroder D.C, Monteiro B.G, Pytlak D.B, de Souza M.C, Mendonca A.J, Ribeiro A.P (2018). Effects of tramadol and acepromazine on intraocular pressure and pupil diameter in young healthy cats. Cien. Rural.

[24] Smith J.D, Ichinose L.Y, Masek G.A, Watanabe T, Stark L (1968). Midbrain single units correlating with pupil response to light. Science.

[25] Bauqioer S.H (2022). Randomised clinical trial comparing the perioperative analgesic efficacy of oral tramadol and intramuscular tramadol in cats. J. Feline Med. Surg.

[26] Makris A, Matala M.E, Tsirigotis A, Karmaniolou I (2012). Apnea and mydriasis after postoperative tramadol administration:An unusual complication and possible underlying mechanisms. Anaesthesia.

[27] Oliva-Domínguez A, Casas-Alvarado A, Miranda-Cortés A.E, Hernández-Avalos I (2021). Clinical pharmacology of tramadol and tapentadol, and their therapeutic efficacy in different models of acute and chronic pain in dogs and cats. J. Adv. Vet. Anim. Res.

[28] Simon B.T, Steagall P.V (2020). Feline procedural sedation and analgesia:When, why and how. J. Feline Med. Surg.

[29] Watanabe R, Monteriro B.P, Ruel H.L.M, Cheng A, Marangoni S, Steagall P.V (2022). The effects of sedation with dexmedetomidine-butorphanol and anesthesia with propofol-isoflurane on feline grimace scale^©^scores. Animals (Basel).

[30] Mahidol C, Niyom S, Thitiyanaporn C, Suprasert A, Thengchaisri N (2015). Effects of continuous intravenous infusion of morphine and morphine-tramadol on the minimum alveolar concentration of sevoflurane and electroencephalographic entropy indices in dogs. Vet. Anaesth. Analg.

[31] Thengchaisri N, Mahidol C (2019). Evaluating the effects of continuous intravenous infusions of tramadol and tramadol-lidocaine on sevoflurane minimum alveolar concentration (MAC) and entropy values in dogs. J. Vet. Med. Sci.

[32] Zaki S, Ticehurst K, Miyaki Y (2009). Clinical evaluation of Alfaxan-CD(R) as an intravenous anaesthetic in young cats. Aust. Vet. J.

[33] Lagos-Carvajal A, Queiroz-Williams P, Cremer J, Pereira C.H.R, Nevarez J, da Cunha A.F, Liu C.C (2020). Effects of a priming dose of alfaxalone on the total anesthetic induction dose for and cardiorespiratory function of sedated healthy cats. Am. J. Vet. Res.

[34] Wheeler E.P, Abelson A.L, Lindsay J.C, Wetmore L.A (2021). Sedative effects of alfaxalone and hydromorphone with or without midazolam in cats:A pilot study. J. Feline Med. Surg.

[35] Rodrigo-Mocholí D, Escudero E, Belda E, Laredo F.G, Hernandis V, Marín P (2018). Pharmacokinetics and effects of alfaxalone after intravenous and intramuscular administration to cats. N. Z. Vet. J.

